# Relationship between serum sodium level and sepsis-induced coagulopathy

**DOI:** 10.3389/fmed.2023.1324369

**Published:** 2024-01-08

**Authors:** Yanyu Han, Jianfeng Duan, Ming Chen, Shijie Huang, Beiyuan Zhang, Yan Wang, Jiali Liu, Xiaoyao Li, Wenkui Yu

**Affiliations:** ^1^Department of Critical Care Medicine, Nanjing Drum Tower Hospital, Drum Tower Clinical College, Nanjing University of Chinese Medicine, Nanjing, China; ^2^Department of Critical Care Medicine, Nanjing Drum Tower Hospital, Affiliated Hospital of Medical School, Nanjing University, Nanjing, China

**Keywords:** sodium, sepsis, coagulation disorders, ICU, hypernatremia

## Abstract

**Purpose:**

A discussion about the correlation between the level of serum sodium and sepsis-induced coagulopathy (SIC).

**Materials and methods:**

A retrospective analysis was conducted on sepsis patients who were admitted to the Intensive Care Unit (ICU) of Nanjing Drum Tower Hospital from January 2021 to December 2022. Based on the presence of coagulation disorders, the patients were divided into two groups: sepsis-induced coagulopathy (SIC) and non-sepsis-induced coagulopathy (non-SIC) groups. We recorded demographic characteristics and laboratory indicators at the time of ICU admission, and analyzed relationship between serum sodium level and SIC.

**Results:**

One hundred and twenty-five patients with sepsis were enrolled, among which, the SIC and the non-SIC groups included 62 and 63 patients, respectively. Compared to patients in the non-SIC group, the level of serum sodium of those in the SIC was significantly higher (*p* < 0.001). Multi-factor logistic regression showed serum sodium level was independently associated with SIC (or = 1.127, *p* = 0.001). Pearson’s correlation analysis indicated that the higher the serum sodium level, the significantly higher the SIC score was (*r* = 0.373, *p* < 0.001). Additionally, the mortality rate of patients with sepsis in the ICU were significantly correlated with increased serum sodium levels (*p* = 0.014).

**Conclusion:**

An increase in serum sodium level was independently associated with an increased occurrence of SIC and also associated with the poor prognosis for patients with sepsis.

## 1 Introduction

Sepsis-induced coagulopathy (SIC) is a significant component of sepsis-related multiple organ dysfunction syndrome (MODS) and is strongly linked to the woresning of microcirculatory issues and tissue organ damage in patients (1–3). The prevalence of SIC in adults ranges from 50 to 70% (4), and it occurs more frequently in sepsis patients compared to those with sepsis-induced acute kidney injury (SAKI) (26–50%) (5–7) and sepsis-induced acute liver injury (SALI) (30%) (8). The development of SIC is primarily associated with the activation of the coagulation pathway, impairment of the anticoagulant system, suppression of fibrinolysis, and platelet aggregation in sepsis patients (9–11). When the organism was infected, inflammatory mediators of pathogen-associated molecular patterns (PAMPs) and pro-inflammatory substances of damage-associated molecular patterns (DAMPs) are synthesized and released into the blood, which puts the organism in a hypercoagulable state (12–14). At this stage, the anticoagulant mechanism is significantly inhibited, which may cause massive microthrombosis and vascular endothelial damage. In the terminal stage, patients may progress to disseminated intravascular coagulation (DIC), which is closely related to the increased mortality rate of patients with sepsis (15, 16). It has been reported that the mortality rate of patients with sepsis combined with DIC is two times greater than that of patients without DIC (17). Any delayed intervention in sepsis-induced coagulation dysfunction may be harmful (18). The International Society on Thrombosis and Hemostasis currently recommends early to identify of coagulation disorders (19).

Sodium ions (Na^+^) are the main cations in extracellular fluids and are important for maintaining extracellular fluid volume, regulating acid–base balance, and maintaining normal osmolality and cellular physiological functions. And it is the most effective of all monovalent cations that activate thrombin (20). Nonetheless, as a result of substantial fluid replacement and increased aldosterone secretion, hypernatremia is also more prevalent among sepsis patients. In a study, the occurrence of hypernatremia in ICU-admitted patients varied from 2 to 6%, while the incidence of ICU-acquired hypernatremia reached as high as 26% (21–23). Moreover, Lindner et al. (22) had shown hypernatremia acquired during the ICU was an independent risk factor for patients death. Hypernatremia is also closely associated with sepsis severity, increased rates of organ failure, and increased in-hospital mortality (24, 25).

Na^+^, a critical thrombin activator, can bind to a specific thrombin site, leading to thrombin activation and stimulation of osmosis (20). These processes regulate the increased expressions of a transcription factor, the nuclear factor of activated T cells 5 (NFAT5), and its binding to the von Willebrand Factor (vWF) promoter, resulting in platelet aggregation (26). Activated thrombin converts fibrinogen into fibrous protein, forming blood clots (27). Additionally, prior research has indicated a connection between serum sodium concentration and damage to vascular endothelial and glycocalyx barriers (17, 28). Excessive sodium concentration leaded to a reduction in the thickness of the endothelial glycocalyx (eGC), a villous layer covering the vascular endothelium (29, 30). However, the integrity of the glycocalyx is important for maintaining normal coagulation function in the body (31).

Presently, there are no clinic studies that have assessed the correlation of serum sodium level with SIC at home and abroad. Thus, this study aimed to retrospectively analyze the relationship between the serum sodium level and SIC in intensive care unit (ICU) patients with sepsis.

## 2 Materials and methods

### 2.1 Clinical information

The clinical data about patients with sepsis who were admitted to the ICU of Nanjing Drum Tower Hospital from January 2021 to December 2022 were retrospectively analyzed. The inclusion criteria were: (1) patients aged ≥18 years; (2) ICU stay ≥24 h; (3) patients conforming to the diagnostic criteria 3.0 for sepsis (confirmed or suspected infection and SOFA score ≥ 2 points) (32). The exclusion criteria were: (1) pregnant patients; (2) patients with a history of chronic liver disease; (3) blood dialysis patients; (4) those with chronic kidney disease; (5) patients with hematological diseases and those taking anticoagulant medications; (6) patients with incomplete clinical data. This study was approved by the Ethics Committee of Drum Tower Clinical Medical College Affiliated with Nanjing University (File Number:2022-038-02).

### 2.2 Data collection

The patient demographic characteristics and laboratory data were collected, based on the patient’s first examination on admission to the ICU. General data: age and gender; underlying diseases: diabetes, hypertension, chronic liver disease, history of chronic kidney disease with hemodialysis, anticoagulation therapy; origins of sepsis; mechanical ventilation (MV); scores: Sequential Organ Failure Assessment (SOFA) score, Acute Physiology and Chronic Health Evaluation (APACHE II) score; died in ICU; laboratory indicators: white blood cell (WBC) count, platelet count (PLT), prothrombin time (PT), activated partial prothrombin time (APTT), international normalized ratio (INR), D-Dimer, fibrinogen (FIB), C-reactive protein (CRP), creatinine (Cr), blood urea nitrogen (BUN), albumin (ALB) level, total bilirubin (TB), serum sodium, serum calcium, serum potassium, and serum phosphorus.

### 2.3 Definition SIC and serum sodium level

The diagnosis of SIC was based on the SIC score, which were assessed using the PT-INR, PLT, and SOFA score. If the sum of points ≥ 4 and the sum of PT-INR and PLT points > 2 were obtained, the patient was diagnosed with SIC (Table 1). the serum sodium concentration was categorized into three groups according to the definitions of previous studies: hypernatremia (>145 mmol/L), normal sodium level (135–145 mmol/L), and hyponatremia (<135 mmol/L) (33, 34).

**Table 1:** Diagnostic scores of SIC.

Categories	0 point	1 point	2 points
PT-INR	≤1.2	>1.2	>1.4
Platelet count (×10^9^/L)	≥150	<150	<100
SOFA score	0	1	≥2

PT, prothrombin time; INR, international standard ratio; SOFA, sequential organ failure score.

### 2.4 Statistical methods

The SPSS 25.0 software was used for statistical analysis. For comparisons between two groups, independent samples *t*-tests were used for measurement data that conformed to normal distribution, expressed as mean ± standard deviation; conversely, nonparametric tests were used, expressed as the median (interquartile spacing). Pearson’s chi-square test or continuous calibration chi-square test was used to compare categorical variables, expressed as percentages (%). We analyzed whether the level of serum sodium was independently associated with SIC by utilizing single- and multifactors logistic regression analysis. We included sex, age, and indicators with *p*-values less than 0.05 in the comparison of baseline data between the two groups in via logistic regression analysis. However, considering the covariance between BUN and Cr, we did not include BUN in the logistic regression analysis. Based on the results we constructed receiver operating characteristic (ROC) curve and calculated the area under the curve (AUC), which was aimed at assessing the efficiency of the serum sodium concentration to predict SIC. One-way ANOVA was used for analysis of variables for more than two groups and for measurement data that conformed to a normal distribution. Correlation between serum sodium levels with SIC scores were analyzed via Pearson’s correlation analysis. Spearman’s correlation analysis was used to process the correlation between serum sodium levels and coagulation parameters The linear-by-linear association was used to analyze whether serum sodium levels were associated with clinical outcomes. A *p*-value < 0.05 for all the statistical results was considered to indicate statistical significance.

## 3 Results

### 3.1 Flowchart of patients meeting inclusion/exclusion criteria for the study

**Figure 1:**
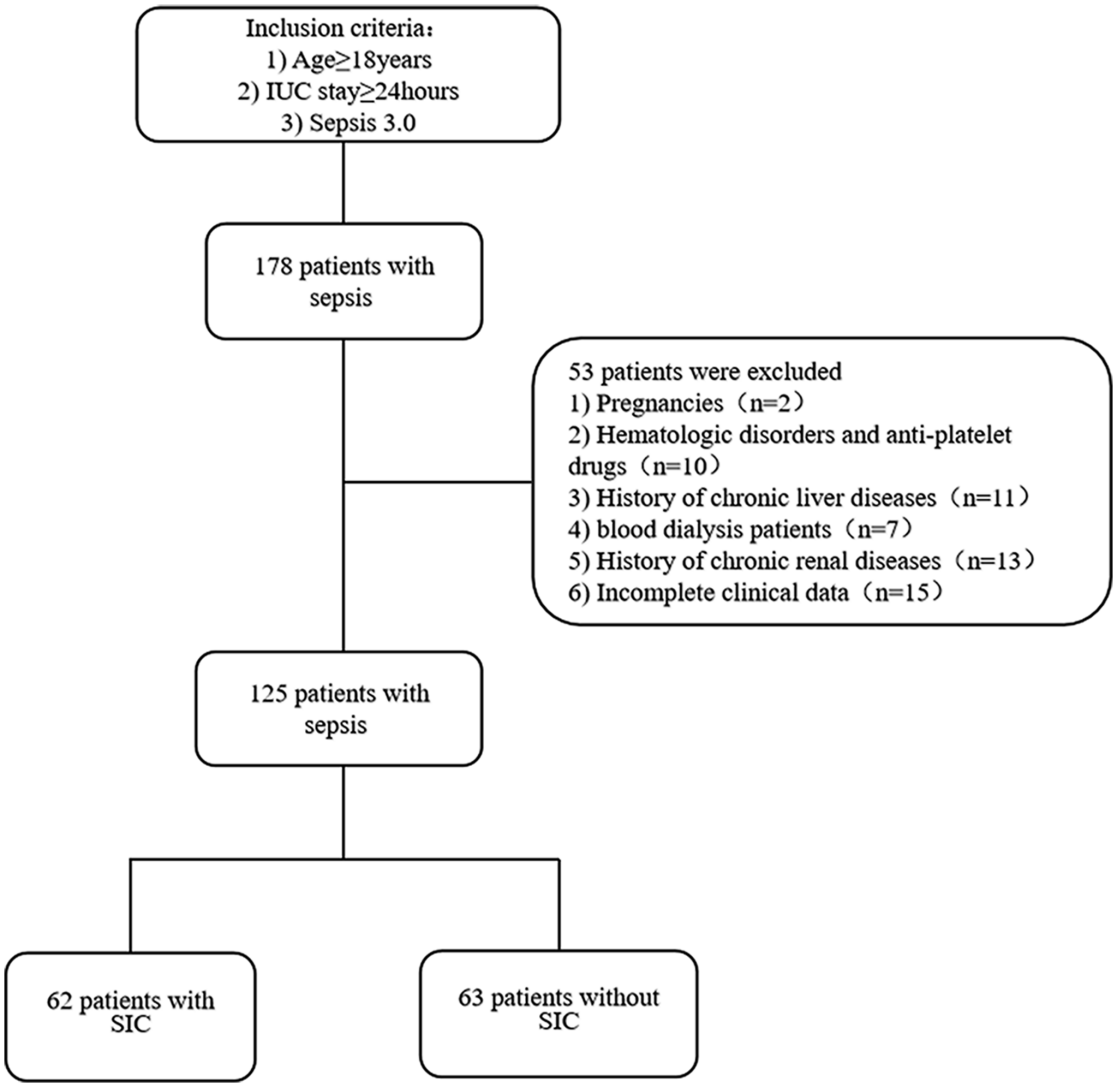
Flow chart of patients with inclusion/exclusion criteria for this study. A total of 178 patients were collected. We excluded 53 patients according to the exclusion criteria. Finally, 125 patients were categorized into SIC (*n* = 62) or non-SIC groups (*n* = 63) using the diagnostic criteria for sepsis-related coagulation disorders.

A total of 178 patients were included, of which, 2 pregnant patients, 10 patients with hematological diseases and taking anticoagulant medications, 11 patients with chronic hypohepatia, 7 blood dialysis patients (of whom 5 had been excluded due to a history of chronic liver and kidney diseases), 13 chronic renal insufficiency patients, and 15 patients with incomplete clinical data were excluded. In the end, the data analysis covered 125 patients in total. Sixty two patients were diagnosed with SIC based on the diagnostic criteria, while 63 patients did not meet the criteria (Figure 1).

### 3.2 Comparison of general information between the SIC and non-SIC groups

The result showed no statistical difference was observed between the two groups in age, gender, underlying diseases, origins of sepsis, needing mechanical ventilation, WBC count, CRP, and ALB levels (*p* > 0.05). However, serum sodium level in the SIC group increased significantly (median, SIC, 144.8 vs. non-SIC, 139.8, *p* < 0.001), while calcium, potassium, and phosphorus levels exhibited no difference between the two groups. Additionally, Cr and TB levels in the SIC group were significantly higher than those in the non-SIC group (median: Cr; SIC, 103.5 vs. non-SIC, 61, *p* = 0.001; TB; SIC, 18.85 vs. non-SIC, 12.6, *p* = 0.014). Moreover, SOFA score and APACHE II score in the SIC group were also significantly higher than those in the non-SIC group (median; SOFA; SIC, 8.5 vs. non-SIC, 6, *p* = 0.003; APACHEII; SIC, 23.95 vs. non-SIC, 20.43, *p* = 0.009), which indicated patients in the SIC group had higher severity (Table 2).

**Table 2:** Comparison of demographic characteristics and laboratory indicators between the SIC and Non-SIC.

Projects	SIC (*n* = 62)	Non-SIC (*n* = 63)	*p*-value
Age, years, mean (SD)	63.1 ± 15.31	60.79 ± 16.98	0.428
Sex, male, *n* (%)	37 (59.70)	40 (63.50)	0.661
SOFA score, median (IQR)	8.5 (5, 13)	6 (4, 8)	0.003^*^
APACHEII score, mean (SD)	23.95 ± 8.47	20.43 ± 6.34	0.009^*^
**Basic disease, *n* (%)**			
Hypertension	24 (38.7)	25 (39.7)	0.911
Diabetes	19 (30.6)	15 (23.8)	0.391
Cardiovascular	9 (14.5)	12 (19)	0.498
Tumor	12 (19.4)	8 (12.7)	0.310
**Origin of sepsis, *n* (%)**			
Lung	28 (45.2)	36 (57.1)	0.180
Abdomen	12 (19.4)	13 (20.6)	0.858
Urinary tract	7 (11.3)	3 (4.8)	0.310
Skin or soft tissue	5 (8.1)	3 (4.8)	0.697
Central nervous system	3 (4.8)	5 (7.9)	0.732
Other	7 (11.3)	3 (4.8)	0.310
Mechanical ventilation, *n* (%)	41 (66.1)	35 (55.6)	0.226
Died in ICU, *n* (%)	24 (38.7)	16 (25.4)	0.111
**Laboratory indicator, median (IQR)**			
WBC (10*9/L)	9.85 (5.53, 15.23)	11 (8.7, 15.4)	0.162
CRP (mg/L)	106.8 (50.30, 196.28)	98.3 (53.4, 161.5)	0.811
Creatinine (μmol/L)	103.5 (61.25, 214.25)	61 (45, 124)	0.001^*^
BUN (mmol/L)	12.4 (8.18, 21.20)	8.5 (6.6, 13.6)	0.002^*^
Total bilirubin (μmol/L)	18.85 (11.73, 37.88)	12.6 (7.9, 26.5)	0.014^*^
Albumin (g/L)	31.05 (29.25, 34.45)	31.4 (29.2, 35.3)	0.925
Serum sodium (mmol/L)	144.8 (140.43, 149.23)	139.8 (136.7, 143.1)	< 0.001^*^
Serum calcium (mmol/L)	2.09 (1.88, 2.20)	2.13 (1.94, 2.27)	0.258
Serum potassium (mmol/L)	4 (3.75, 4.60)	4.02 (3.65, 4.29)	0.174
Serum phosphorus (mmol/L)	1.125 (0.73, 1.39)	0.89 (0.73, 1.17)	0.142

SOFA, Sequential Organ Failure Assessment; APACHEII, Acute Physiology and Chronic Health Status Assessment System II; WBC, white blood cell count; CRP, C-reactive protein; BUN, blood urea nitrogen; *p < 0.05.

### 3.3 Relationship between serum sodium level and SIC

The age and sex, as well as SOFA score, Cr, TB, and Na^+^ level, were included in the single-factor logistic regression analysis. These indicators were then included in a multi-factor logistic regression analysis. The results showed that Cr (OR, 1.006; 95% CI, 1.010 ~ 1.001; *p* = 0.011) and serum sodium level (OR, 1.127; 95% CI, 1.051 ~ 1.208; *p* = 0.001) were independently correlated with SIC (Table 3).

**Table 3:** Logistic regression analysis of coagulation dysfunction associated with sepsis.

Variables	Single-factor logisitic analysis		Multi-factor logisitic analysis	
	OR value (95% CI)	*p* value	OR value (95% CI)	*p* value
Ages	1.009 (0.987 ~ 1.031)	0.424		
Sex	0.851 (0.414 ~ 1.751)	0.661		
SOFA score	1.149 (1.052 ~ 1.254)	0.002*		
APACHEII score	1.066 (1.014 ~ 1.121)	0.012*		
Creatinine (μmol/L)	1.006 (1.002 ~ 1.010)	0.006*	1.005 (1.001 ~ 1.010)	0.012*
Total bilirubin (μmol/L)	1.006 (0.995 ~ 1.016)	0.297		
Serum sodium (mmol/L)	1.121 (1.054 ~ 1.193)	< 0.001*	1.125 (1.050 ~ 1.207)	0.001*

SOFA, Sequential Organ Failure Assessment; APACHEII, Acute Physiology and Chronic Health Status Assessment System II; *p < 0.05.

### 3.4 ROC curve analysis of serum sodium level to predict SIC

**Figure 2:**
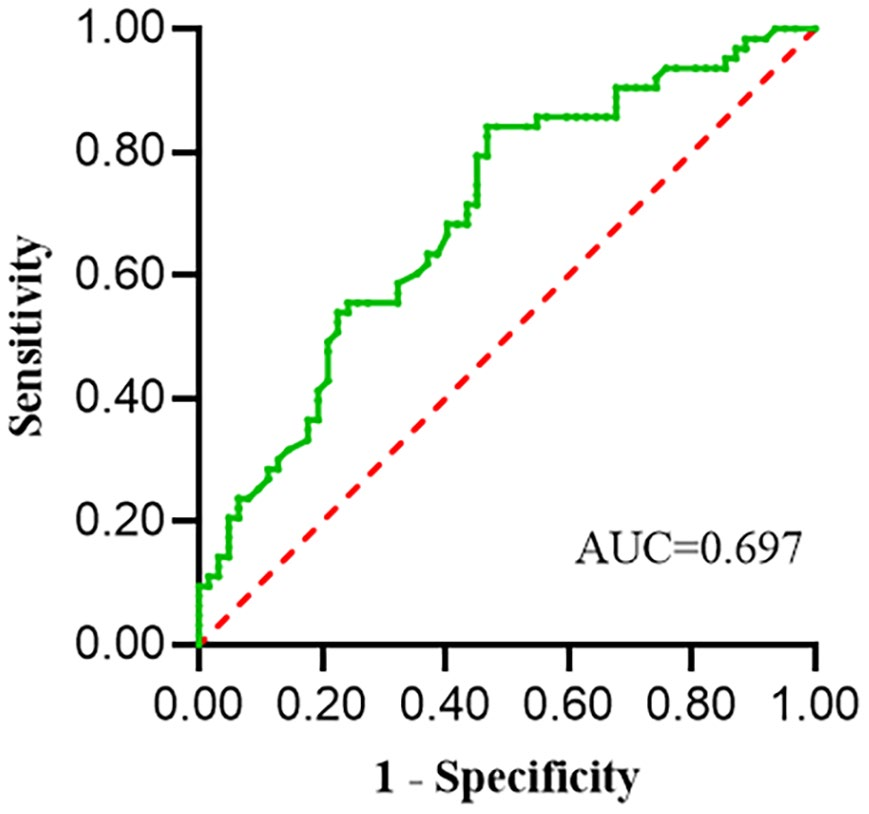
ROC curve analysis of serum sodium levels to predict SIC. The sensitivity and specificity of serum sodium to predict SIC were 53.2 and 84.1%, respectively; the critical value was 144.65 mmol/L; the area under the curve was 0.697 (*p* < 0.001); 95% confidence interval: 0.605–0.789.

We conducted a ROC curve. The result showed that serum sodium level had a predictive value for the occurrence of SIC (AUC = 0.697, 95% CI, 0.605–0.789, *p* < 0.001). The best cut-off value for predicting SIC was 144.65 mmol/L, with a sensitivity of 53.2% and a specificity of 84.1% (Figure 2).

### 3.5 Correlation between serum sodium level and SIC score

**Figure 3:**
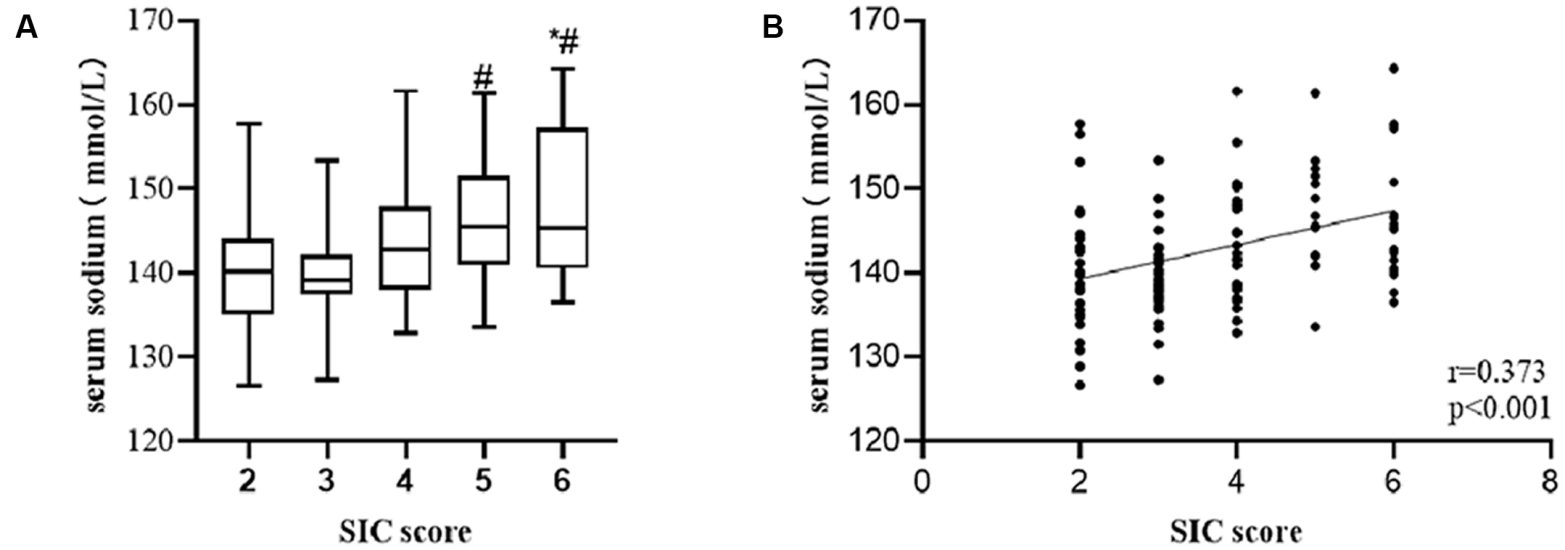
Among patient with sepsis, serum sodium level was significant higher in patients in the group with higher SIC score compared to those in the group with lower SIC score **(A)** (*p-value *<* 0.05, comparison vs. 2 points group; #*p*-value < 0.05, comparison vs. 3 points group). There was a positive correlation of serum sodium level with SIC score **(B)**.

According to the SIC scores, we created 5 groups (2 points, 3 points, 4 points, 5 points, and 6 points) and compared differences in serum sodium among groups. We also analyzed the correlation between serum sodium level and SIC score. Based on the results, we drew a boxplot and a scatter diagram. The number of patients in each group was 27, 36, 28, 15, and 19, respectively. Compared to the 5 and 6 points groups, the level of serum sodium in 3 points group was significantly lower (mean, 5 points group, 146.41 vs. 3 points group, 143.27, *p = 0.026*; 6 points group, 147.53 vs. 3 points group, 143.27, *p* = 0.002), whereas the 2 points group exhibited a statistical difference when compared to serum sodium level in 6 points groups (mean, 6 points group, 147.53 vs. 2 points group, 139.99, *p* = 0.009) (Figure 3A). There was a correlation between serum sodium level and SIC score (*r* = 0.373, *p* < 0.001) (Figure 3B). The higher the SIC score, the higher the serum sodium level.

### 3.6 Correlation between serum sodium level and coagulation parameters

**Figure 4:**
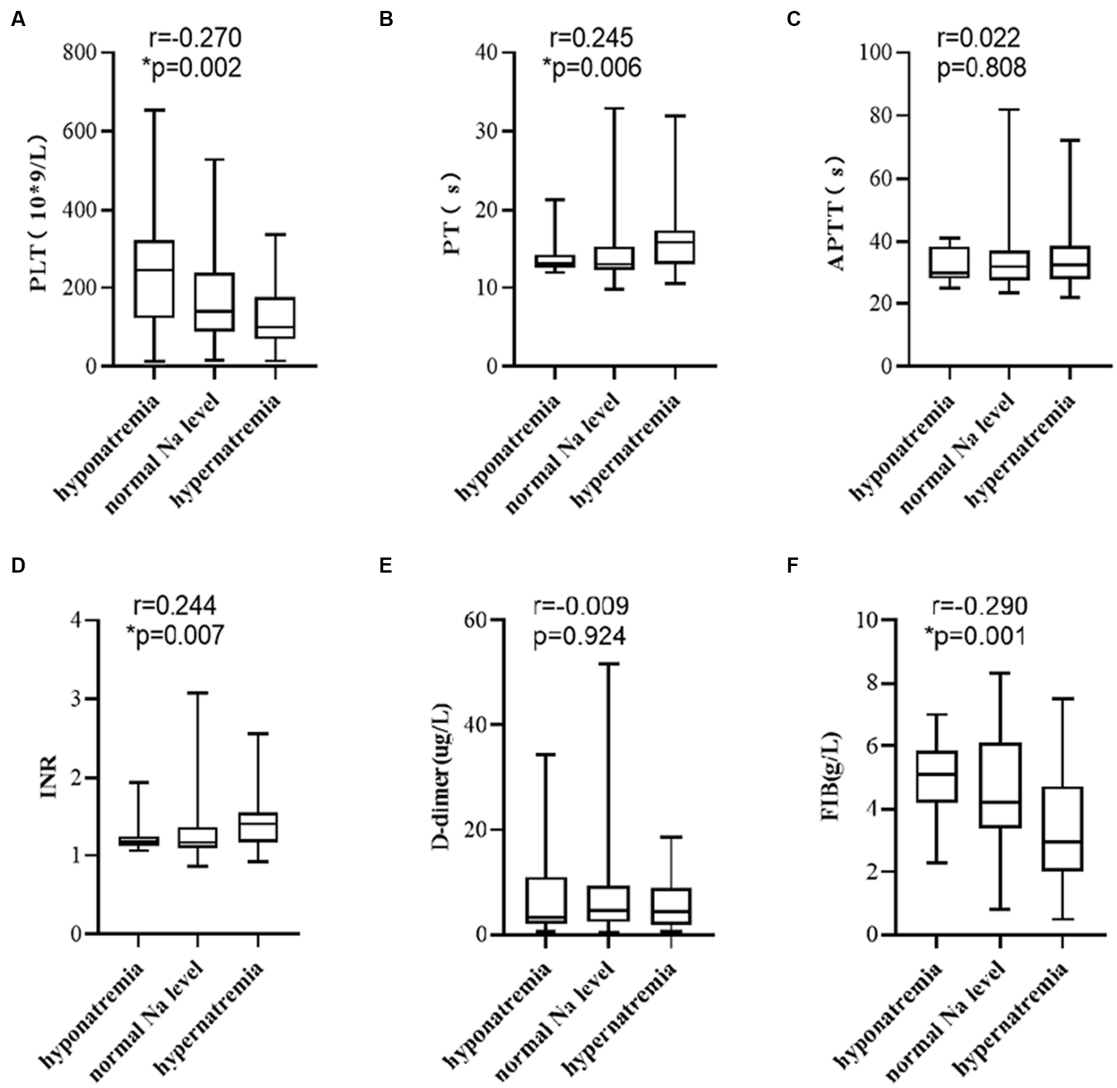
Elevated serum sodium level was associated with **(A)** PLT, **(B)** PT, **(D)** INR, and **(F)** FIB, but not with **(C)** APTT, and **(E)** D-dimer. *p*-values by Spearman’s correlation analysis test.

Based on the diagnostic criteria of hypo- and hypernatremia, 125 patients were divided into hyponatremia, normal Na level, and hypernatremia groups. The number of patients in each group was 13, 74, and 38, respectively. A correlation analysis was conducted. The results showed higher serum sodium level displayed a significant correlation with lower PLT level (Figure 4A, *r* = −0.270, *p* = 0.002), higher PT level (Figure 4B, *r* = 0.245, *p* = 0.006), higher INR level (Figure 4D, *r* = 0.244, *p* = 0.007), and lower FIB level (Figure 4F, *r* = −0.290, *p* = 0.001), but not with APTT (Figure 4C, *r* = 0.022, *p* = 0.808) and D-dimer levels (Figure 4E, *r* = -0.009, *p* = 0.924).

### 3.7 Correlation of serum sodium level with clinical outcomes

**Figure 5:**
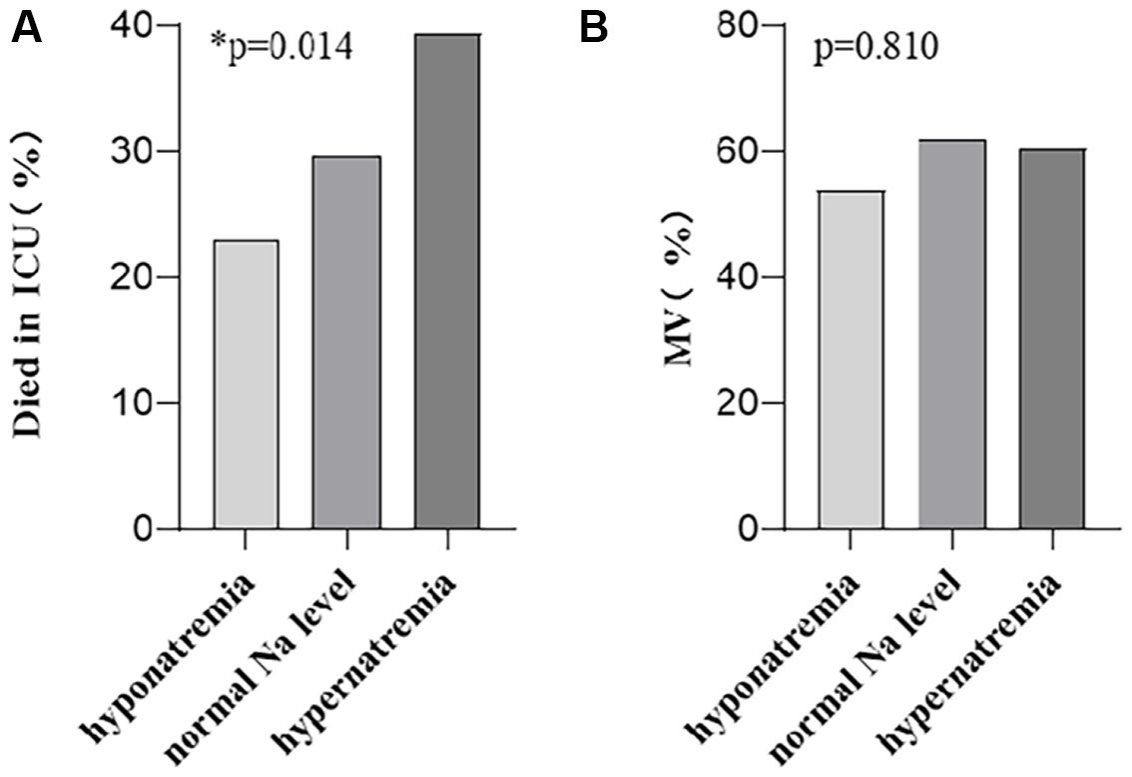
Higher serum sodium levels were associated with **(A)** an increased in-ICU mortality rate, but not with **(B)** the needing of mechanical ventilation therapy. *p*-values by linear-by-linear association test.

The linear-by-linear association was used to evaluate whether the clinical outcomes of patients with sepsis correlate with the serum sodium level. The patients were divided into hyponatremia, normal Na level, and hypernatremia groups as per the serum sodium levels. The in-ICU mortality of each group was 3, 22, and 15, while the number of patients needing mechanical ventilation in each group was 7, 46, and 23, respectively. Our results showed that the in-ICU mortality rate of patients with sepsis correlated with an increased serum sodium level (Figure 5A, *p* = 0.014), but not with mechanical ventilation (Figure 5B, *p* = 0.810).

## 4 Discussion

In this study, we explored the correlation between the level of serum sodium and the SIC. We analyzed the relationship between serum sodium and SIC score. The present study showed a positive correlation between serum sodium levels and SIC scores. An increase in serum sodium levels was independently associated with the development of SIC.

Although there are no studies on the relationship between serum sodium and coagulation disorders, studies have shown that serum sodium concentrations are related to the eGC. The serum sodium concentration in the body plays an important role in maintaining eGC stability. The eGC, a layer of negatively charged villus-like structures covering the endothelial surface of blood vessels, attracts circulating Na + ions in the vascular cava (35) and plays a beneficial role in sodium buffering *in vivo* (36). Consequently, the glycocalyx serves as a significant extrarenal regulator of extracellular sodium and serve as a reservoir for substantial sodium storage (37). When the body experiences sodium overload, it disrupts Na + homeostasis. This alteration causes a transition in endothelial cells from releasing sodium to absorbing sodium (28), leading to damaged in vascular endothelium. This, in turn, results in a reduction in the release of nitric oxide (NO). NO can dilate blood vessels, inhibit platelet activation, prevent platelet aggregation, adhesion, and prevent thrombosis (38, 39). Martin et al. (17) subjected human umbilical vein endothelial cells (HUVECs) to sodium (Na) concentrations of 134 mEq/L (control medium), 150 mEq/L, and 160 mEq/L, respectively. Then they found that excessive sodium concentrations all resulted in a significant increase in the shedding of eGC damage markers. They also measured glycocalyx thickness and found that the thickness of the cellular glycocalyx was significantly reduced by a factor of two under the Na 160 mEq/L concentration compared to the control group under Na 134 mEq/L. A study by Zheng et al. (40) reported that, compared to the normal chow (NC) diet group, the NC diet with 4% salt (NC4%) induced microcirculatory disturbances and glycocalyx degradation in mice. Glycocalyx damage is closely associated with the development of coagulation dysfunction, which has also been reported in several papers (41–43). These findings may laterally indicate that elevated serum sodium levels impair eGC, which in turn affects coagulation.

We investigated the relationship between serum sodium levels and coagulation parameters. The findings revealed that the higher serum sodium levels were associated with activation of the coagulation state, primarily manifesting as reduced platelet counts, prolonged PT, increased INR, and diminished FIB levels. Various mechanisms have been proposed to elucidate the coagulation dysfunction that could be related to increased serum sodium. The blood coagulation factor Xa (FXa) is an important serine protease in the coagulation cascade that plays a vital role in physiological hemostasis. However, excessive thrombin levels lead to the transformation of soluble fibrinogen into the insoluble fibrous protein, thus, resulting in thrombus formation (44–47). Relative studies suggested that thrombin displays better catalysis in the process of clotting in the presence of Na^+^ (48–51). Rezaie and He (52) also proved that Na^+^ can effectively activate thrombin which may relate to that it can bind to the 225 s loop residue of Try conformation of FXa. Moreover, Dmitrieva and Burg (26) cultured HUVECs in a high-sodium environment with different osmotic pressures and found that the secretion of vWF displayed a sodium-dependent increase while the high-sodium environment stimulated increased NFAT5 production. The vWF is secreted by endothelial cells and can bind to blood platelets, which is crucial for thrombus formation; the increased NFAT5 activity also contributes to increased vWF production in endothelial cells. Furthermore, Dmitrieva and Burg (26) revealed that when compared to the renal cortex, the vWF proteins and interstitial sodium chloride levels in the renal medulla were significantly higher. This indicated that the elevation of extracellular sodium within the physiological range is sufficient to increase vWF levels, thereby enhancing its coagulation ability and the risk of thrombus formation. Moreover, we found that the increased serum sodium levels were related to the decreased blood platelets and fibrinogen levels, which was consistent with the above-mentioned studies.

We also analyzed the relationship between serum sodium level and clinical outcomes in patients with sepsis. The results showed that the higher the serum sodium level was, the greater the mortality rate was for patients with sepsis in the ICU. In addition, the ROC curve showed that a serum sodium concentration of 144.65 mmol/L had predictive value for the occurrence of SIC. Li et al. (53) found that higher serum sodium level was associated with an increased mortality rate in patients with sepsis in a large-sample, multicenter study. Thongprayoon et al. (54) also reported that borderline hypernatremia (143–147 mmol/L) was associated with an increased hospital mortality rate in a study on serum sodium and the risk of death in hospital patients. All of these support the finding that elevated serum sodium levels are associated with severity in septic patients.

In addition, another new finding of our study was that Cr was also independently associated with SIC. However, the association between Cr and coagulation has not been determined. Cr serves as a predictor of renal function. The kidney is one of the organs most likely to be involved when an organism suffers from an infection. Therefore, we speculate that Cr may be associated with sepsis complicated by acute kidney loss. One study showed that coagulation function was significantly abnormal in patients with SAKI compared with patients without AKI, as evidenced by thrombocytopenia, elevated INR, and prolonged PT (55). This may be related to the fact that coagulation activation crosstalks with an inflammatory response to form extensive microthrombi, resulting in renal ischemic injury (56).

To the best of our knowledge, this study is not only the first study to investigate the correlation between serum sodium level and coagulation dysfunction in sepsis patients, but also the first study on the relationship between serum sodium level and coagulation function. However, this study has several limitations: first, it was a retrospective single-center study with a small sample size; second, our patient population was skewed toward elderly patients; and third, the causality and mechanism of action could not be proven. Therefore, a large sample size is needed to validate our findings further. Further studies are needed in the future to reveal the specific mechanism of action involved in the relationship between serum sodium level and SIC.

In conclusion, our retrospective analysis results suggested that an increase in the serum sodium level was independently associated with an increased occurrence of SIC and was also associated with an increase in-ICU mortality rate in septic patients. Higher serum sodium levels may lead to glycocalyx injury and exacerbate coagulation dysfunction. Therefore, we should pay attention to the serum sodium level in sepsis patients and further explore the molecular mechanisms underlying the relationship between the serum sodium and coagulation function to provide potential targets for improving coagulation function in sepsis patients.

## Data availability statement

The raw data supporting the conclusions of this article will be made available by the authors, without undue reservation.

## Ethics statement

The studies involving humans were approved by Ethics Committee of Nanjing Drum Tower Hospital, The Affiliated Hospital School of Nanjing University Medical School, Nanjing, China. The studies were conducted in accordance with the local legislation and institutional requirements. Written informed consent for participation was not required from the participants or the participants’ legal guardians/next of kin because this is a retrospective analysis.

## Author contributions

YH: Conceptualization, Methodology, Visualization, Writing – original draft. JD: Conceptualization, Methodology, Visualization, Writing – original draft. MC: Supervision, Visualization, Writing – review & editing. SH: Data curation, Writing – review & editing. BZ: Data curation, Writing – review & editing. YW: Data curation, Writing – review & editing. JL: Data curation, Writing – review & editing. XL: Project administration, Writing – review & editing. WY: Project administration, Writing – review & editing.
